# Trichostatin A relieves anxiety-and depression-like symptoms in APP/PS1 mice

**DOI:** 10.3389/fphar.2024.1333235

**Published:** 2024-03-20

**Authors:** Qiang Su, Yu-Hua Ren, Guo-Wei Liu, Yan-Ping Gao, Jiu-Xuan Zhang, Jin-Nan Zhang, Xia-Xia Pei, Tian Li

**Affiliations:** ^1^ Department of Laboratory Medicine of Fenyang College, Shanxi Medical University, Fenyang, Shanxi, China; ^2^ Department of Physiology, School of Basic Medicine, Key Laboratory of Cellular Physiology, Ministry of Education, Shanxi Key Laboratory of Cell Physiology, Shanxi Medical University, Taiyuan, Shanxi, China

**Keywords:** Trichostatin A, Alzheimer’s disease, APP/PS1 mice, anxiety, depression, CST7

## Abstract

**Background:**

Cognitive deficits and behavioral disorders such as anxiety and depression are common manifestations of Alzheimer’s disease (AD). Our previous work demonstrated that Trichostatin A (TSA) could alleviate neuroinflammatory plaques and improve cognitive disorders. AD, anxiety, and depression are all associated with microglial inflammation. However, whether TSA could attenuate anxiety- and depression-like behaviors in APP/PS1 mice through anti-inflammatory signaling is still unclearly.

**Methods:**

In the present study, all mice were subjected to the open field, elevated plus maze, and forced swim tests to assess anxiety- and depression-related behaviors after TSA administration. To understand the possible mechanisms underlying the behavioral effects observed, *CST7* was measured in the hippocampus of mice and LPS-treated BV2 microglia.

**Results:**

The results of this study indicated that TSA administration relieved the behaviors of depression and anxiety in APP/PS1 mice, and decreased *CST7* levels in the hippocampus of APP/PS1 mice and LPS-induced BV2 cells.

**Conclusion:**

Overall, these findings support the idea that TSA might be beneficial for reducing neurobehavioral disorders in AD and this could be due to suppression of *CST7*-related microglial inflammation.

## 1 Introduction

Alzheimer’s disease (AD) is the most common type of neurodegenerative dementia in the elderly, with a rising rate worldwide (Scheltens et al., 2021). AD is characterized by the extraneuronal deposition of amyloid-β plaques and intraneuronal aggregation of hyperphosphorylated tau protein, and activation of multiple neuroinflammatory pathways that, ultimately, lead to neuronal death (Self and Holtzman, 2023). Although the core AD clinical manifestations are declined in memory and cognition, the presence of neuropsychiatric symptoms such as anxiety and depression are commonly observed in 80% of AD cases (Zhao et al., 2016). A number of studies have investigated anxiety and depression in association with AD. The prevalence of anxiety in mild-to-severe AD is around 39%, and almost 40% of patients with mild-to-moderate AD suffer from depression (Zhao et al., 2016). Further, the frequent overlap between anxiety and depression is especially in patients with mild AD. On the one hand, anxiety and depression generally appear early in the course of AD, representing a risk factor for the course or development of AD. On the other hand, evidence is growing that anxiety and depression and AD share some common biological basis. Therefore, anti-depression and anti-anxiety treatments are considered as promising strategy to alleviate AD (Cacabelos et al., 2023).

In the nervous system, one of the best characterized histone modifications is the acetylation of N-terminal lysine residues that shifts the conformation of chromatin into a relaxed state, leading to gene transcription (Park et al., 2022). Histone deacetylases (HDACs) are capable of regulating chromatin function through histone acetylation and play essential roles in learning and memory, and synaptic plasticity. Moreover, histone acetylation has also been implicated in anxiety and depression pathophysiology. Clinical and preclinical investigations have shown an increase in HDACs expression paralleled by histone acetylation reduction in depressive patients and animals (Covington et al., 2009; Hobara et al., 2010; Golden et al., 2013; Borba et al., 2021; Park et al., 2021). Similar phenomena are also found in the autopsy of anxiety patients and animals (Sah et al., 2019; Peng et al., 2021). Moreover, several studies show that histone deacetylase inhibitor (HDACi) can ameliorate anxiety- and depression-like behavior in patients and rodents (Sah et al., 2019; Peng et al., 2021; Choi, 2022; Baek et al., 2023). Taken together, these findings suggest that is closely involved in the pathophysiology of anxiety and depression is closely related to histone acetylation. Trichostatin A (TSA) is a classical and widely used HDACi inhibitor, which inhibits both class I and class II HDAC enzymes (Yoshida et al., 1990). Montagud-Romero et al. reported that TSA reversed anxiety-like symptoms and memory impairments, and decreased activity levels of the HDAC4 in the hippocampus in mice with maternal binge alcohol drinking (Montagud-Romero et al., 2019). Kimijima et al. found that TSA alleviated the emotional abnormality induced by maladaptation to stress in mice (Kimijima et al., 2022). Recently, we observed the ability of TSA, to reverse the hypoacetylation of histone H4 (H4K12) in the hippocampus and increase albumin expression and Aβ clearance, as well as ameliorate Alzheimer’s disease-related pathology and cognitive deficits in APP/PS1 mice (Su et al., 2021). However, the role of TSA on anxiety and depressive behavior in AD mice and its underlying molecular mechanisms remains unexplored.

Here, we set out to investigate the effects of TSA on anxiety- and depression-like behavior in APP/PS1 transgenic AD mouse model. We first performed elevated cross maze and forced swimming tests in mice to show that TSA indeed ameliorated anxiety- and depression-like behavior in APP/PS1 mice. We then compiled data from published datasets to show that Cystatin-F (*CST7*) is the common gene between disease-associated microglia (DAM) genes and depression-associated (DA) genes. *CST7* plays key role in immune regulation and was upregulated in AD-related DAM (Chen et al., 2020). DAM is identified a unique subtype of microglia, and implicated in mediating pathogenesis of AD (Keren-Shaul et al., 2017). Moreover, we also demonstrated that the expression level of *CST7* was significantly upregulated in the hippocampus of APP/PS1 mice, while that was suppressed with TSA. These data are consistent with previous research shown that *CST7* is upregulated in AD (Rasmussen et al., 2022; Baleviciute and Keane, 2023). We further investigated this *in vitro* and showed that TSA reduced LPS-induced *CST7* expression in BV2 cells. These data bring important insight to the functional role of TSA, relieving anxiety and depression, and present that the mitigating effect of TSA on anxiety and depression relevant to AD might via extenuate *CST7*-related microglial inflammation.

## 2 Methods

### 2.1 Animals

**FIGURE 1:**
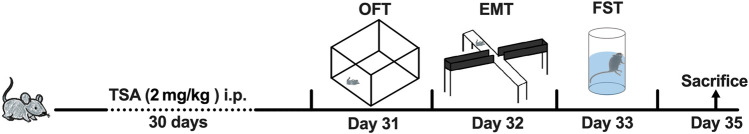
The scheme of TSA administration and behavioral tests. OFT, open field test; EMT, elevated plus maze test; FST, forced swimming test.

Male APPswe/PS1dE9 (APP/PS1) mice and wild-type (WT) littermates were obtained from the Model Animal Research Center of Nanjing University (Nanjing, China). Mice were housed in 12:12 h light/dark cycle animal room (temperature 23°C ± 1 °C; humidity 40%–50%; *ad libitum* food and water). The 8-month-old mice (n = 37) were randomly divided into four groups: WT + Vehicle (n = 9), WT + TSA (n = 9), APP/PS1 + Vehicle (n = 10), and APP/PS1 + TSA (n = 9). Based on our previous study (Su et al., 2021), TSA (T6270, TargetMol) was solubilized in 100% dimethylsulfoxide (DMSO) and then diluted with normal saline to a final concentration of 0.2 mg/mL. TSA (2 mg/kg) or equivalent vehicle (solvent of TSA) was administered via i. p. injection daily for 30 days before behavioral tests (Figure 1). All animal experimental procedures were performed in accordance with relevant guidelines and regulations of the Institutional Animal Care Committee of Shanxi Medical University.

### 2.2 Open field test (OFT)

To assess locomotor activity and disinhibition-like behavior, mice were subjected to the open field test (Li et al., 2018). The apparatus consisted of a white box (40 × 40 × 50 cm) with overhead video recording. Each mouse was gently placed in the center of the apparatus and allowed to explore the field freely for 5 min. Animal activity was recorded using an automated tracking system (SMART 3.0 software, RWD Life Science). The total distance and time spent in the center of the field were processed and analyzed. The test box was sanitized after each trial using a 70% ethanol spray.

### 2.3 Elevated plus maze test (EMT)

The elevated plus maze test is a widely used behavioral assay to detect anxiety-like behavior in rodents (Walf and Frye, 2007). A plus-shaped apparatus elevated 50 cm above the floor composed of two opposite closed arms opposite to two open arms was used for the test. Mice were placed in the central zone toward the open-arm direction and given a maximum of 5 min to freely explore the apparatus. The time explored the open arms and the number of entries in the open arms were recorded and quantified. After each trial, the maze was sanitized using 70% ethanol spray.

### 2.4 Forced swimming test (FST)

To measure depressive-like behavior in mice, the forced swim test was used as previously described (Kraeuter et al., 2019). Mice were individually placed into the transparent cylinders (height: 25 cm, diameter: 10 cm), filled with water (depth of water: 15 cm and temperature: 25 ± 1 °C) and allowed to freely swim for 6 min. The immobility time of the mouse was recorded and measured during the last 4 min.

### 2.5 Identification of the common gene

Disease-associated microglia (DAM) genes were obtained from Keren-Shaul et al.‘s research (Keren-Shaul et al., 2017), including *APOE*, *CST7*, *LPl*, *SPP1*, *ITAGX*, *CLEC7A*, *CSF1*, *IGF1*, *AX1*, *ANK*, *TYROBP*, *CD63*, *CTSD*, *CTSB*, *TREM2*, *CTS1*, *CD9*, *CTSZ*, *CCL6*, *P2RY12* and *TMEM119*, which are highly significant genes related to DAM. Depression-associated (DA) genes were collected from Gao et al.‘s research (Gao et al., 2018), containing *UQCRC1*, *GZMB*, *NDUFB9*, *NSF*, *SLC17A5*, *CTSH*, *NDUFB10*, *UQCR10*, *ATOX1*, *CST7* and *CTSW*, which are the key genes for the pathogenesis of major depression. Through Venny diagrams (Venny 2.1 software; https://csbg.cnb.csic.es/BioinfoGP/venny.html), we then assessed the common dysregulated gene in DAM genes and DA genes.

### 2.6 Western blotting

The total protein extracted from the hippocampus was used for Western blotting, and the method was described previously (Li et al., 2022). The primary antibodies used were *CST7* (orb101860, Biorbyt) and β-actin (D191047, Sangon Biotech). The membranes were incubated in primary antibodies at 4°C overnight, followed by an incubation with HRP-conjugated secondary antibodies at room temperature for 2 h. Protein bands were detected using ECL Western blot Detection kit (P0018FS, Beyotime), and the images were captured using Azure c300 Chemiluminescent Western blot Imaging System (Azure Biosystems). The band intensity was analyzed with AlphaView SA (Fluorchem FC3, ALPHA).

### 2.7 Cell culture and reagents preparation

Murine BV2 microglia cells were bought from the cell bank (Chinese Academy of Medical Sciences), and cultured in DMEM media supplemented with 10% fetal bovine serum (FBS, ExCell) and antibiotics (100 U/mL penicillin and 100 μg/mL streptomycin) at 37 °C in a humidified 5% CO_2_ incubator. For Western blot, cells were seeded at a proper density into a 6-well plate and exposed to LPS (100 ng/mL; Solarbio) with or without TSA (125 nM) or ITSA (50 μM; S8323, Selleck). treated under the indicated conditions for 24 h, and then the protein extract was collected.

### 2.8 Statistics

SigmaPlot 12.3 was used to analyze the data by two-way ANOVA or one-way ANOVA followed by the appropriate *post hoc* test. All data were presented as means ± standard error (SEM) with *p* < 0.05 considered as statistically significant.

## 3 Results

### 3.1 TSA mitigated disinhibition-like behavior in APP/PS1 mice

**FIGURE 2:**
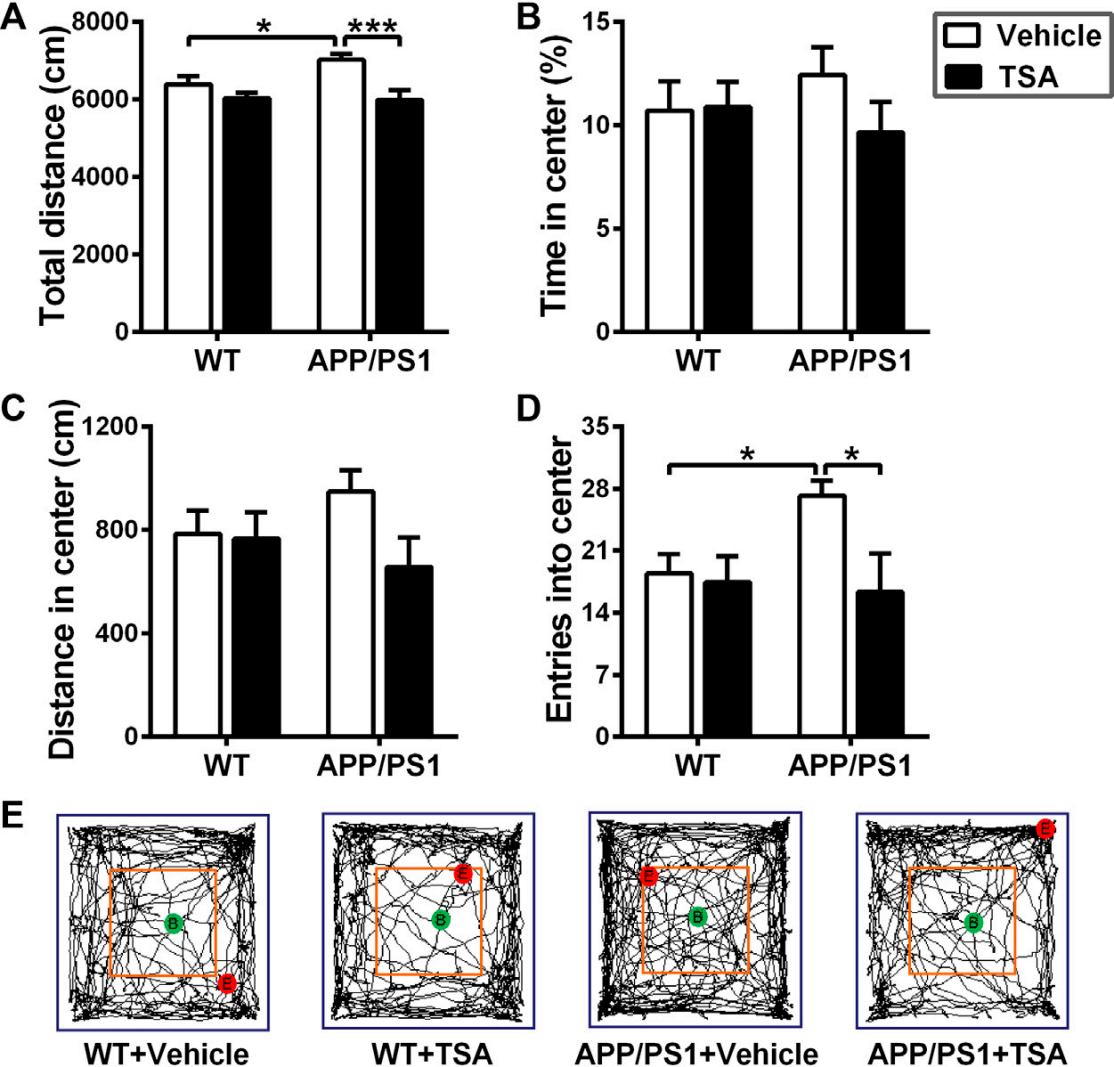
Treatment with TSA relieved disinhibition-like behavior in APP/PS1 mice. **(A)** Total distance traveled in the open field arena. **(B)** Time spent in the central area of open field. **(C)** Distance moved in the central area of open field. **(D)** Entries into the central area of open field. **(E)** Representative trajectory plots of mice exploring an open field arena. n = 9-10. *p < 0.05, ***p < 0.001.

Abnormal locomotor activity and disinhibition-like behavior are among the common comorbidities in individuals with AD as well as animal models of AD (Jenkins et al., 2022; Wang et al., 2022). In the open field test, total distance traveled significantly increased in APP/PS1 mice as compared to WT mice (*p* < 0.05), while that was markedly decreased in TSA-treated APP/PS1 mice (*p* < 0.001) (Figure 2A). Moreover, it was evident that the number of entries in the central area was greater in APP/PS1 mice than in WT mice (*p* < 0.05), whereas that was notably reduced in APP/PS1 mice treated with TSA (*p* < 0.05) (Figure 2D). Although there was a tendency to increase in distance moved and time spent in the central area in APP/PS1 mice in comparison with WT mice, it could be observed that TSA treatment alleviated these increases in APP/PS1 mice (Figures 2B,C). These results suggested that TSA alleviated disinhibited exploratory behavior and decreased locomotor activity in APP/PS1 mice.

### 3.2 TSA treatment ameliorated anxiety-like behavior in APP/PS1 mice during the elevated plus maze test

**FIGURE 3:**
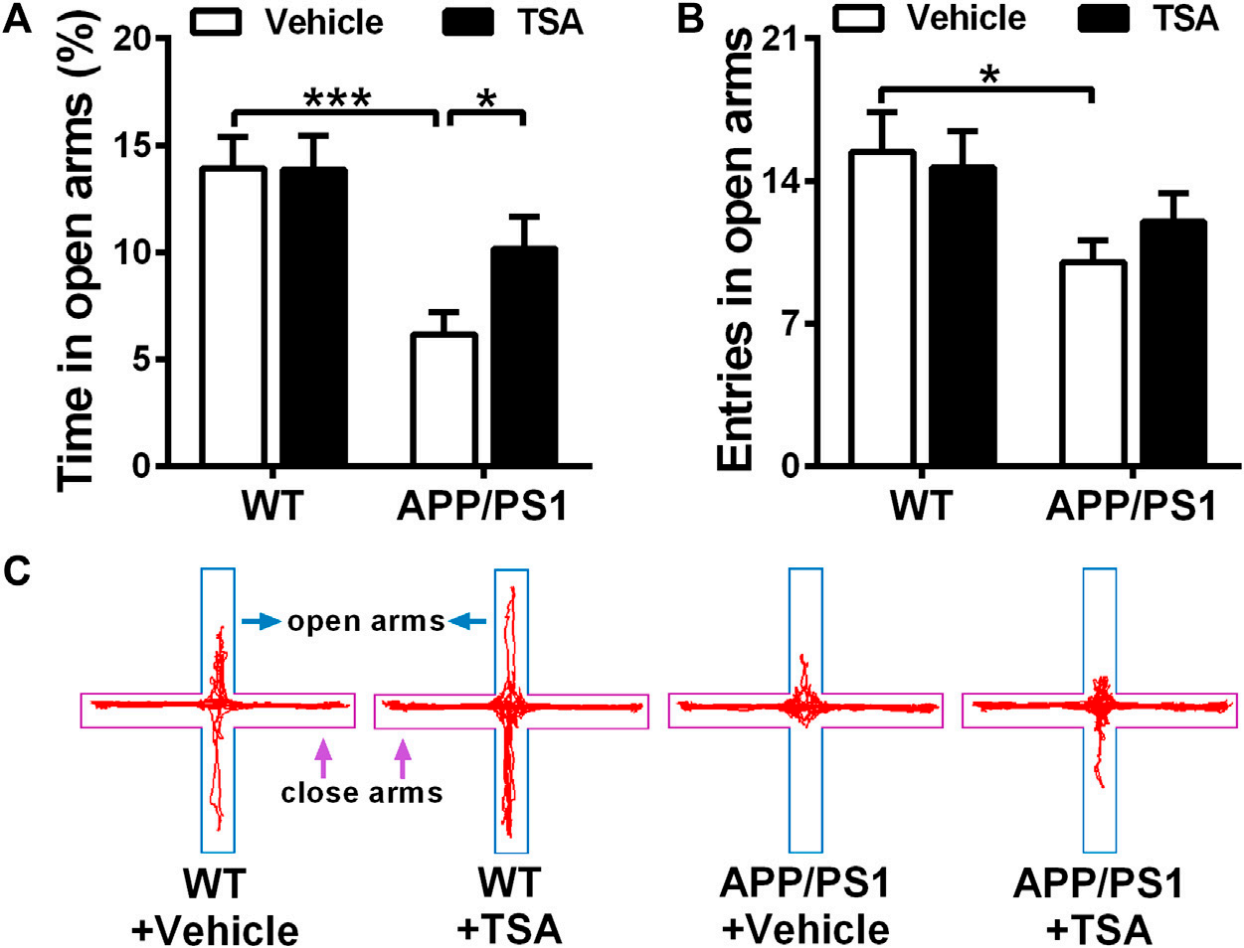
APP/PS1 mice with TSA treatment showed a significant improvement in anxiety-like behavior. **(A)** Time spent in the open arms and **(B)** The number of entries entered the open arms of each group in the elevated plus maze. **(C)** Representative traces of mice in EPM. n = 9-10. *p < 0.05, ***p < 0.001.

Next, we evaluated whether TSA could attenuate anxiety-like behaviors in APP/PS1 mice. Elevated plus maze results showed that in comparison with WT mice, APP/PS1 mice spent significantly less time in the open arms (*p* < 0.001). However, the treatment of APP/PS1 mice with TSA notably prolonged time explored in the open arms compared to vehicle-treated APP/PS1 mice (*p* < 0.05) (Figure 3A). Additionally, the number of open arm entries was significantly lower in vehicle-treated APP/PS1 mice than that WT mice (*p* < 0.05), whereas administering TSA to APP/PS1 mice resulted in a slight increase in the number of open arm entries compared to vehicle-treated APP/PS1 mice (Figure 3B). These results indicated that TSA showed a remarkable antianxiety effect in APP/PS1 mice.

### 3.3 TSA attenuated depression-like behavior of APP/PS1 mice in the forced swimming test

**FIGURE 4:**
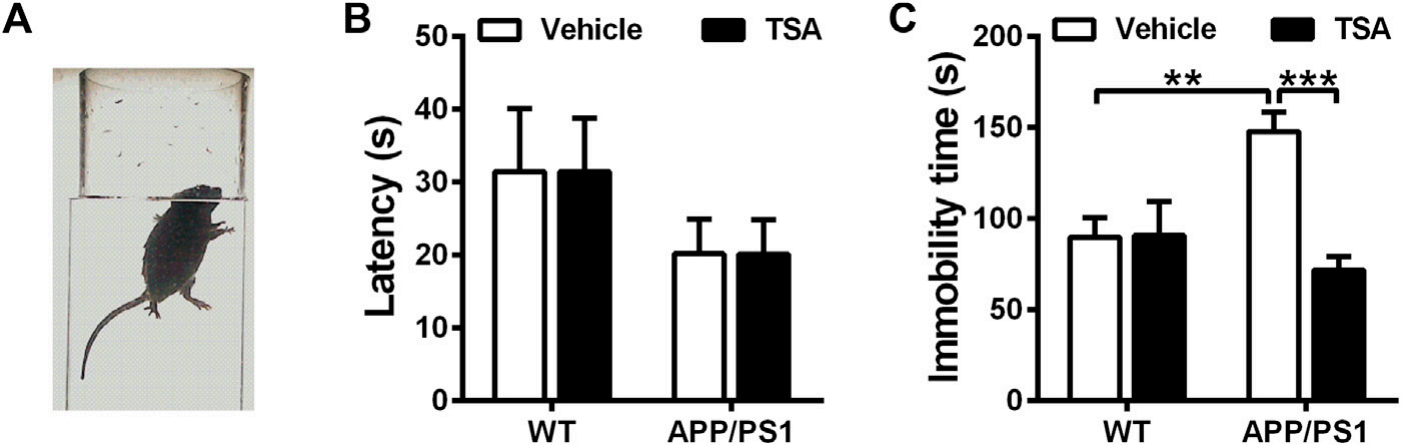
TSA reduced the immobile time of APP/PS1 mice. **(A)** Schematic showing forced swimming test. **(B)** Latency to the first immobility in forced swimming test. **(C)** The immobile time during the last 4 min in the forced swimming test. n = 9-10. **p < 0.01, ***p < 0.001.

To further evaluate the role of TSA on neurobehavioral disorders, we performed the forced swimming test to detect the effect of TSA on depressive behavior in APP/PS1 mice. In the forced swimming test, the latency to first immobility was comparable among the four groups. As shown in Figure 4C, APP/PS1 mice displayed a notable increase in the immobile time as compared with WT mice in the forced swimming test (*p* < 0.01), while APP/PS1 mice accepted TSA treatment spent less immobile time than vehicle-treated APP/PS1 mice (*p* < 0.001). These results indicated that TSA alleviated depression-like behavior in APP/PS1 mice.

### 3.4 TSA down-regulated *CST7* expression in the hippocampus of APP/PS1 mice and LPS-induced BV2 cells

**FIGURE 5:**
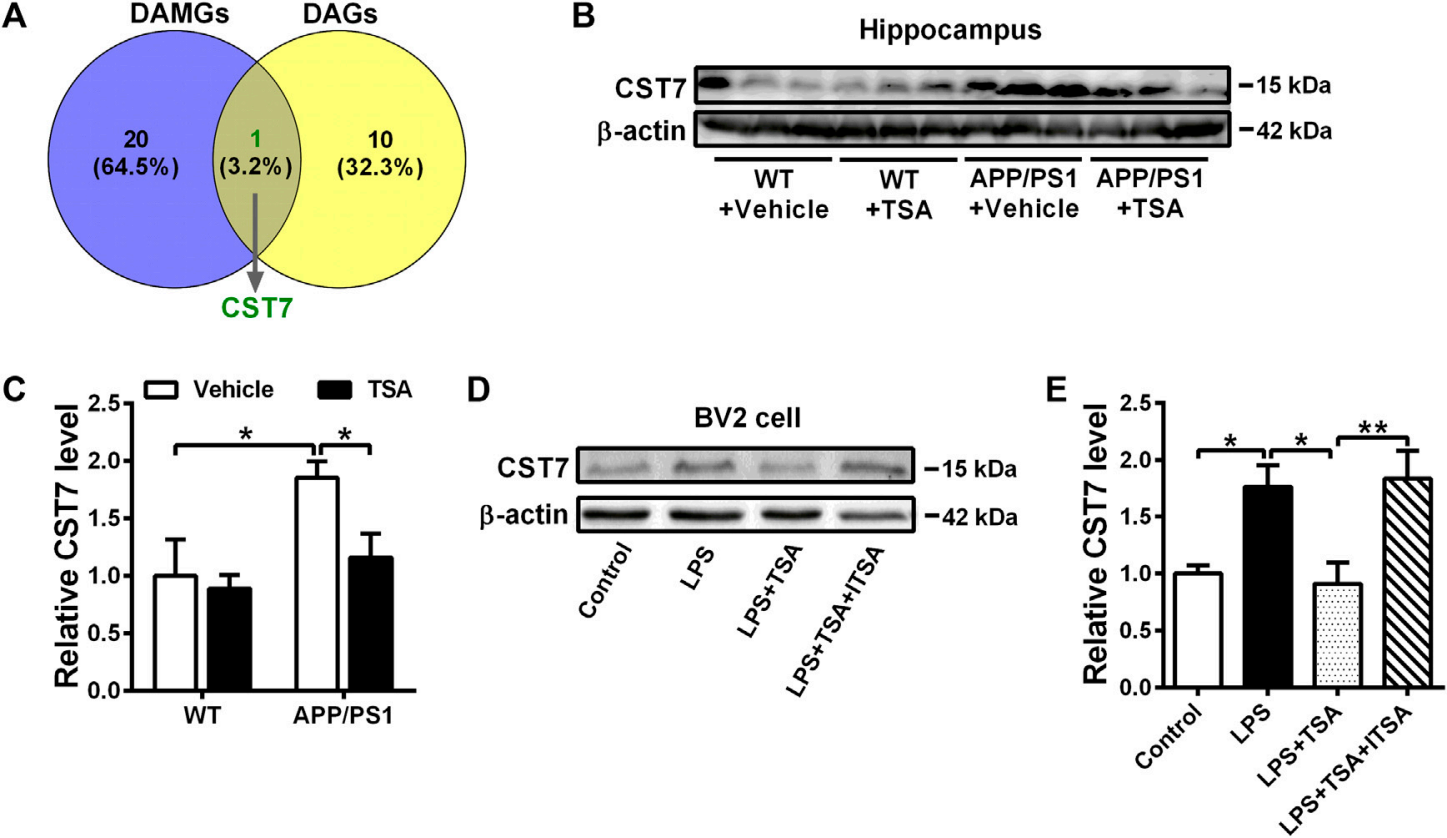
TSA prevents the increment of *CST7* in the hippocampus of APP/PS1 mice and LPS-induced BV2 cells. **(A)** The Venn diagram indicates an overlap of DEGs between DAM genes and DA genes. **(B, D)** Representative Western blot images of *CST7* and β-actin from the hippocampus of mice **(B)** and BV2 cells **(D)**. **(C, E)** Levels of *CST7* were measured in the hippocampus of mice **(C)** and BV2 cells **(E)**. Relative levels were obtained as a ratio of *CST7*/β-actin and then normalized to WT + Vehicle group or Control group. n = 3. *p < 0.05, **p < 0.01.

It has been reported that microglial inflammation plays a key role in the pathogenesis of AD and neuropsychiatric symptoms, such as depression (Wang et al., 2018b), while its underlying molecular mechanisms are still unclearly. Hence, to determine potential specific differentially expressed genes (DEGs) that are responsible for microglial inflammation between AD and depression, we first analyzed the overlap of common DEGs between disease-associated microglia (DAM) genes (Keren-Shaul et al., 2017) and depression-associated (DA) genes (Gao et al., 2018) using the Venn function, and found the common gene, *CST7*, which is an endogenous inhibitor of cysteine proteases. Next, to verify if the expression of *CST7* was differentially modulated in the hippocampus of the different experimental groups, we measured the levels of *CST7* by Western blot analysis. As shown in Figure 5B, C, the expression of *CST7* in the hippocampus of vehicle-treated APP/PS1 mice was notably higher than that in vehicle-treated WT mice (*p* < 0.05). Nevertheless, TSA administration restored near-normal levels of *CST7* in the hippocampus of APP/PS1 mice (*p* < 0.05). Correspondingly, we also found that there was a significant increase in *CST7* expression level in LPS-treated BV2 cells compared to the control group (*p* < 0.05). However, a significant decrease was observed in TSA-treated conditions (*p* < 0.05), which could be reversed by ITSA administration (*p* < 0.01). These data indicated that TSA attenuated depression-like behavior in APP/PS1 mice possibly via inhibiting *CST7*-related microglial inflammation.

## 4 Discussion

AD is one of the most common neurodegenerative disorders in older adults. Although progressive memory loss and cognitive decline are the predominant symptoms of AD, neuropsychiatric symptoms are the frequent prodromal symptoms or comorbid condition in over 90% of AD patients (Scheltens et al., 2016), causing a serious burden for patients’ families and the society. It is noteworthy that anxiety and depression are the most common neuropsychiatric disorders in AD. Epidemiological studies have reported that anxiety in mild-to-severe AD is around 39%, and almost 40% of patients with mild-to-moderate AD suffer from depression (Zhao et al., 2016). Therefore, anxiety and depression as the risk factors for AD have been widely concerned. However, the majority of AD research is focused on cognitive decline, insufficient attention has been paid to neuropsychiatric symptoms in AD, and a lack of effective pharmacological therapies.

Our previous study found that TSA has therapeutic potential against AD, and confirmed that TSA effectively alleviated cognitive behavior and neuropathology in APP/PS1 animal model of AD (Su et al., 2021). TSA, a natural hydroxamic acid extracted from *Streptomyces* hygroscopicus, is a potent and specific reversible HDACi (Yoshida et al., 1995). TSA is capable of inhibiting the activity of HDAC by chelating a zinc ion in the active-site pocket through its hydroxamic acid group, and has been demonstrated to upregulate cellular histone acetylation, induce cell differentiation, promote transcription and expression of transcription factors, and regulate related signaling pathways (Williams, 2001). Accumulating evidence have shown that HDAC expression has been implicated in various neuropsychiatric symptoms including anxiety and depression, suggesting that histone hypoacetylation and HDAC plays an adjustive role in the negative affective symptoms. Different levels of increases in HDAC2 and HDAC5 levels are found in the periphery and brain of depressed patients and depressed model animals (Iga et al., 2007; Chen et al., 2019; Rey et al., 2019; Seo et al., 2021). HDACi has been utilized to treat psychic disorders in both preclinical and clinical pharmacological studies. Azargoonjahromi reported that valproic acid/sodium valproate (VPA), an HDACi, has anxiolytic effects in patients with anxiety disorders (Azargoonjahromi, 2023). Moreover, Gurbani et al. showed that belinostat, an HDACi with blood–brain barrier permeability, significantly improved depressive symptoms in patients with glioblastoma compared with control subjects (Gurbani et al., 2019). Studies have shown that TSA reverses anxiety-like symptoms and memory impairments induced by maternal binge alcohol drinking in mice (Montagud-Romero et al., 2019), and also involved in the regulation of central 5-HT1A receptor expression (Zhu et al., 2021) and BNDF expression (Montagud-Romero et al., 2019) associated with adjustment of anxiety- and depressive-related behaviors. These studies provide an insight that TSA has the potential to improve neuropsychiatric disorders. APP/PS1 transgenic mice are an internationally recognized mice model for AD, and display the core pathological processes found in AD, including neuroinflammatory plaques, which mainly are induced by microglial activation, and exhibit both cognitive behavioral impairments and neuropsychiatric disorders relevant to AD, including memory loss, hyperlocomotion, anxiety-like behavior and depression-like behavior. However, to date, the effect of TSA on anxiety- and depression-like behavior in AD has not been elucidated.

Transgenic AD mouse models are typical early-onset AD (EOAD) models, and their development has been beneficial to examine the molecular basis of AD and speed-up the discovery of therapeutic targets. Unlike EOAD, genetic susceptibility to late-onset AD (LOAD) is more complex with variations in many genes significantly associated with increased risk of varying degree. As a result, developing an animal model that simulates late-onset AD has always been controversial. The neuropathology observed in late onset AD (LOAD) is essentially identical to that observed in EOAD (Walsh and Selkoe, 2004), although there may be significant differences between EOAD and LOAD. Due to the complexity of the LOAD model and its consistency with EOAD pathology, transgenic AD mice are currently the most important animal model for studying AD pathology and developing therapeutics and preventatives. Due to the complexity of the LOAD model and its consistency with EOAD pathology, transgenic AD mice are currently the most important animal model for studying AD pathology and developing therapeutics and preventatives. In the present study, APP/PS1 transgenic mouse model was used. APP/PS1 transgenic mouse models overexpress the human mutant forms of APP and PS1, and exhibit age-dependent aggravation in amyloid pathology and cognitive impairments similar to that observed in human AD brains. Moreover, they exhibit plaque-associated dystrophic neurites, microglial activation, synaptic impairments and deficits in synaptic plasticity. Hence, APP/PS1 transgenic mouse model has been widely recognized as a chronic AD animal model. In order to investigate the role of TSA in neuropsychiatric behavior in APP/PS1 mice, open field, elevated plus maze, and forced swimming tests were administered in the present study. The results from the open field test revealed that the APP/PS1 mice showed longer distance traveled, more entries and time spent in the central area compared to WT mice, indicating that APP/PS1 mice displayed increased locomotor activity. Our results are consisted with other researchers, their data also presented that APP/PS1 mice behaved disinhibition and ran aimlessly in the open field, especially exhibited increased the time percentage in center area of the open field, indicating that APP/PS1 mice were nervous and anxious after entering the new environment (Wang et al., 2020; Wang et al., 2022; Gao et al., 2023). However, we found that TSA-treated APP/PS1 mice took shorter total distance traveled, as well as fewer entries and time in the central area *versus* vehicle-treated APP/PS1 mice. These findings provide evidence that TSA therapy contributes to attenuating locomotor activity and disinhibition-like behavior in APP/PS1 mice, suggesting that TSA might have a positive effect on improving neuropsychiatric disorders in AD mice. These results are in line with the above-mentioned studies that TSA has the effect of antagonizing neuropsychiatric disorders (Montagud-Romero et al., 2019; Zhu et al., 2021). Thus, we speculated that TSA might also relieve anxiety and depressive behavior in APP/PS1 mice. We further investigate the effect of TSA on anxiety- and depression-like behavior in APP/PS1 mice via the elevated plus maze and forced swimming tests. Our data revealed that APP/PS1 mice displayed anxiety-like behavior with less time spent in open arms and reduced entries entered open arms, whereas that was remarkably reversed after TSA administration. In addition, the results of FST also presented that the immobile time was noticeably declined in APP/PS1 mice with TSA treatment. On the basis of our results, we concluded that TSA intervention relieved anxiety- and depression-like behavior in APP/PS1 mice. Combined with our previous findings, the improvement of TSA in anxiety- and depression-like behavior might also be another essential factor in alleviating cognitive impairment and AD progression. Certainly, it cannot be ruled out that TSA the mitigation effect of TSA on anxiety- and depression-like behaviors in APP/PS1 mice may also be attributed to the remission of brain pathologies. As mentioned by Zhu and Montagud-Romero above, TSA might also improve the neuropsychiatric behavior of AD mice through neuroprotection. Additionally, Hsing et al. and Kannan et al. have reported that TSA could suppress the inflammatory response in microglia (Kannan et al., 2013; Hsing et al., 2015), which might be related to reversing neuropsychiatric.

A growing body of research has shown that microglia-mediated neuroinflammation is associated with the etiology of neuropsychiatric disorders, such as anxiety and depressive disorders (Frick et al., 2013; Han et al., 2020; Ji et al., 2022). Notably, neuroinflammation has a prominent role in the pathogenesis of AD, especially microglia as major players in neuroinflammation. Accordingly, we focus on seeking the pathological changes of microglia in the brain of neuropsychiatric diseases and AD. Our previous study confirmed that TSA treatment decreased the number and area of microglia in the hippocampus of APP/PS1 mice, and observed that the ratio of activated BV2 cells to branched resting BV2 cells decreased significantly (Su et al., 2021). Additionally, Hsing et al. and Fleiss et al. demonstrated that TSA alleviated microglial inflammation *in vivo* and *in vitro* (Fleiss et al., 2012; Hsing et al., 2015). Furthermore, using cellular transcriptomics, disease-associated microglia (DAM) as a unique microglia-type was discovered to play an irreplaceable role in the pathogenesis of AD, and predicts many new sites of AD risk, which may have important implications for the pathogenesis and treatment of AD (Keren-Shaul et al., 2017). Similarly, Gao et al. identified several differential genes that are associated with depression by using the bioinformatic analysis (Gao et al., 2018). We then employed Venn analysis to identify common differential genes implicated in AD, depression, and anxiety. Surprisingly, we discovered that the two gene sets share *CST7* (encoding protein Cystatin F), an endogenous inhibitor of cysteine proteases, which is associated with microglial inflammation in the brain. In our study, we showed that the expression of *CST7* in the brains of APP/PS1 mice was evidently increased compared with WT mice, while that was notably decreased after TSA treatment, suggesting that the alleviation of anxiety- and depression-like behaviors in APP/PS1 may also benefit from TSA due to its inhibition of *CST7* expression in their brains. To further verify this, we performed *in vitro* experiments and found that *CST7* expression was markedly increased after LPS intervention, but decreased by TSA, which was in agreement with the results *in vivo*. Thus, we hypothesized that TSA might alleviate anxiety- and depression-like behaviors in APP/PS1 mice by inhibiting *CST7*-related microglial inflammatory response. This finding is consistent with our previous data that TSA treatment decreased the number and area of microglia in the hippocampus of APP/PS1 mice and suppressed inflammation in BV2 cells (Su et al., 2021). A key finding of our study was that high levels of microglial *CST7* could be implicated in the depressive-like behavior associated with AD, and that TSA could attenuate neuropsychiatric disorders in AD by inhibiting microglial inflammatory response linked to *CST7*. However, the underlying molecular mechanisms of TSA on inhibiting *CST7* expression also need to be further explored. Although some researcher found that TSA may improve the inflammatory response through the FoxO3a signaling pathway (Shen et al., 2022). Wang et al. suggested that TSA and its analogues might produce anti-microglial inflammation through the Akt-RhoGTPase pathway (Wang et al., 2018). It is still unclear that the molecular mechanism TSA decreases inflammation.

Taken together, these findings provide evidence that TSA had beneficial effects on improving neuropsychiatric behavior in APP/PS1 mice, and this could be due to inhibition of CST7-related microglial inflammation. TSA might offer a new promising avenue in AD treatment.

## Data Availability

The raw data supporting the conclusion of this article will be made available by the authors, without undue reservation.
