# Supplementary material for: Trichostatin A relieves anxiety-and depression-like symptoms in APP/PS1 mice
Source: Front Pharmacol. 2024 Mar 20;15:1333235. doi: 10.3389/fphar.2024.1333235 (PMC10987769; doi:10.3389/fphar.2024.1333235)

Hippocampus

CST7

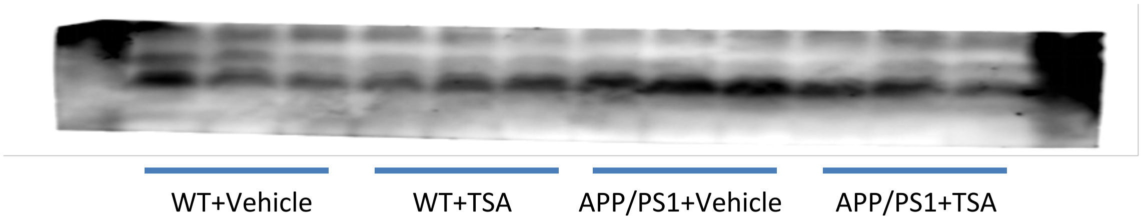

β-actin

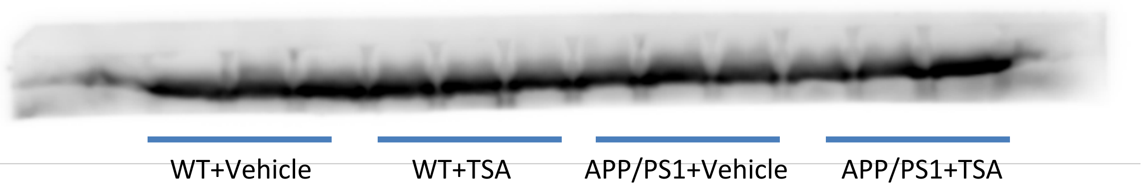

BV2 cell

CST7

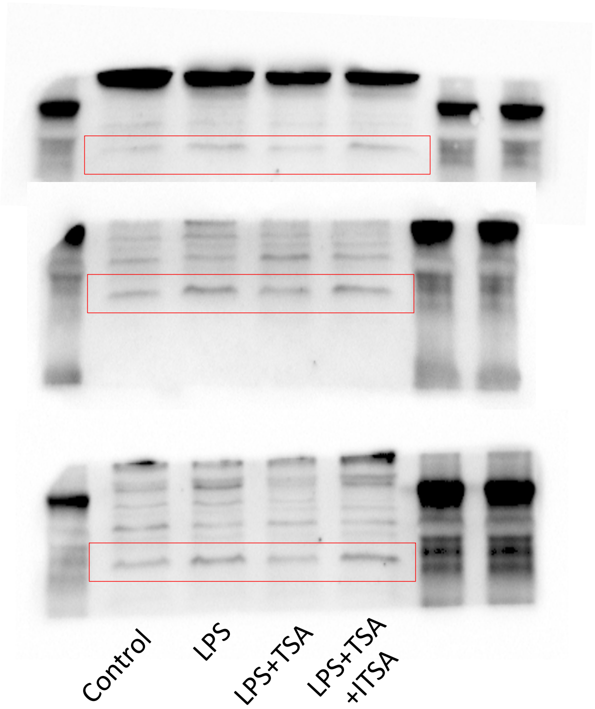

β-actin

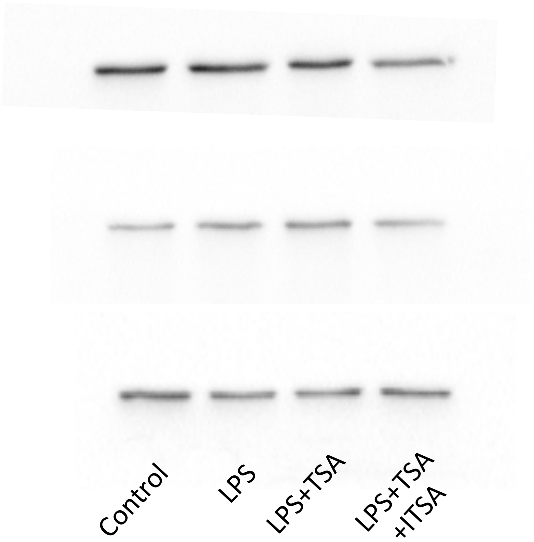

Supplement: Supplementary file 3 [file DataSheet1.PDF]
